# Efficacy, durability, and safety of faricimab in patients from Asian countries with neovascular age-related macular degeneration: 1-Year subgroup analysis of the TENAYA and LUCERNE trials

**DOI:** 10.1007/s00417-023-06071-8

**Published:** 2023-06-09

**Authors:** Kanji Takahashi, Chui Ming Gemmy Cheung, Tomohiro Iida, Timothy Y. Y. Lai, Masahito Ohji, Yasuo Yanagi, Mika Kawano, Shino Ohsawa, Tomoyuki Suzuki, Aachal Kotecha, Hugh Lin, Vaibhavi Patel, Balakumar Swaminathan, Won Ki Lee, On behalf of the TENAYA, LUCERNE Investigators

**Affiliations:** 1 Department of Ophthalmology, Kansai Medical University, School of Medicine, 2-5-1 Shinmachi, Hirakata City, Osaka 573-1191 Japan; 2 Singapore Eye Research Institute, Singapore National Eye Centre, Duke-NUS Medical School, National University of Singapore, Singapore, Singapore; 3 Tokyo Women’s Medical University, Tokyo, Japan; 4 Department of Ophthalmology and Visual Science, The Chinese University of Hong Kong, Hong Kong, New Territories China; 5 Department of Ophthalmology, Shiga University of Medical Science, Otsu, Shiga Japan; 6 Department of Ophthalmology and Microtechnology, Yokohama City University, Yokohama, Japan; 7 Chugai Pharmaceutical Co., Ltd., Tokyo, Japan; 8 Roche Products Ltd., Welwyn Garden City, UK; 9 Genentech, Inc., South San Francisco, CA USA; 10 F. Hoffman-La Roche Ltd., Mississauga, Canada; 11 Nune Eye Hospital, Seoul, South Korea

**Keywords:** Angiopoietin-2, Asia, Faricimab, Neovascular age-related macular degeneration, Vascular endothelial growth factor-A, Vascular stability

## Abstract

**Purpose:**

To evaluate 1-year efficacy, durability, and safety of faricimab among patients from Asian countries in the TENAYA/LUCERNE trials of neovascular age-related macular degeneration (nAMD).

**Methods:**

Treatment-naïve patients with nAMD were randomly assigned (1:1) to faricimab 6.0 mg up to every 16 weeks (Q16W), based on disease activity at weeks 20 and 24, or aflibercept 2.0 mg Q8W. The primary endpoint was change in best-corrected visual acuity (BCVA) from baseline averaged over weeks 40, 44, and 48.

**Results:**

In the pooled TENAYA/LUCERNE trials, there were 120 (9.0%) and 1209 (91.0%) patients in the Asian (faricimab *n* = 61; aflibercept *n* = 59) and non-Asian country (faricimab *n* = 604; aflibercept *n* = 605) subgroups, respectively. In the Asian country subgroup, mean BCVA change from baseline at the primary endpoint visits was 7.1 (95% CI, 4.3–9.8) letters with faricimab and 7.2 (4.4–10.0) letters with aflibercept. In non-Asian country patients, mean vision gains were 6.1 (5.2–7.1) and 5.7 (4.8–6.7) letters with faricimab and aflibercept, respectively. At week 48, 59.6% of Asian country patients in the faricimab group achieved Q16W dosing (vs. 43.9% non-Asian) and 91.2% achieved ≥ Q12W dosing (vs. 77.5% non-Asian). Central subfield thickness reductions were similar between the subgroups, with meaningful and similar reductions from baseline observed at the primary endpoint visits and over time. Faricimab was well tolerated in both subgroups, with an acceptable safety profile.

**Conclusion:**

Consistent with the global TENAYA/LUCERNE findings, faricimab up to Q16W showed sustained visual and anatomical benefits in patients with nAMD from Asian and non-Asian countries.

**Trial registration:**

ClinicalTrials.gov identifier: NCT03823287 (TENAYA); NCT03823300 (LUCERNE). Date of registration: January 30, 2019.

**Supplementary Information:**

The online version contains supplementary material available at 10.1007/s00417-023-06071-8.

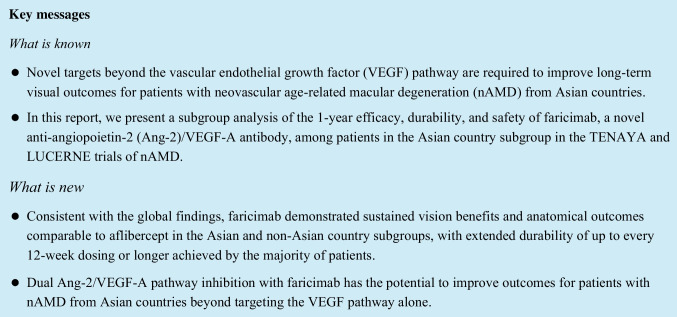



## Introduction

Neovascular age-related macular degeneration (nAMD) remains a leading cause of vision impairment and blindness in adults aged ≥ 50 years worldwide [1, 2]. The prevalence of early and late AMD is reported to be 6.8% and 0.56%, respectively, in the Asian population [3]. Because Asia accounts for more than 60% of the world population, it is expected that Asia will have the largest prevalence of AMD globally by 2040, with a projected 113 million cases [4]. Hence, optimizing treatment strategies and approaches for Asian patients with nAMD is of clear importance.

The introduction of intravitreal anti–vascular endothelial growth factor (VEGF) therapy has greatly improved visual outcomes for patients with nAMD and is now the standard of care [5–9]. However, vision gains achieved in clinical trials are not always realized in clinical practice, at least in part due to undertreatment associated with the need for frequent intravitreal injections and monitoring visits to maintain clinical benefits, which is burdensome for patients [10–13]. This has been demonstrated for both American and European populations [10–13], as well as in the Asian population [14–16]. Therefore, there is a significant need for additional strategies to reduce treatment burden and optimize treatment outcomes, including new therapies targeting additional pathways beyond VEGF alone, for Asian patients with nAMD.

The angiopoietin-tyrosine kinase with immunoglobulin-like and epidermal growth factor homology domains (Ang-Tie) pathway plays a key role in regulating vascular stability and inflammation under physiological and pathological conditions [17]. In homeostatic vasculature, Ang-1–mediated activation of the Tie2 receptor promotes endothelial cell survival, pericyte recruitment, and improved endothelial barrier function, thereby maintaining vessel stability and preventing leakage and inflammation [17]. During disease states, including nAMD, an angiogenic switch occurs, resulting in Ang-2 overexpression relative to Ang-1 [18, 19]. Under these conditions, Ang-2 acts as a competitive antagonist of Ang-1 by binding to Tie2 without inducing its activation [20]. This disruption to Ang-1/Tie2 signaling results in vascular instability through pericyte dropout, increased inflammation, and weakening of endothelial cell junctions [20]. In addition, Ang-2 promotes vascular responsiveness to the angiogenic effects of VEGF-A, enhancing its effects on vascular permeability and neovascularization [21]. Together, these findings support the hypothesis that dual Ang-2/VEGF pathway inhibition may reduce vascular leakage and inflammation and improve outcomes for patients over VEGF inhibition alone [22].

Faricimab, the first bispecific antibody designed for intraocular use via intravitreal injection, inhibits 2 distinct pathways by independently binding and neutralizing both Ang-2 and VEGF-A [22]. The phase III TENAYA (NCT03823287) and LUCERNE (NCT03823300) trials reported the efficacy, durability, and safety of faricimab up to every 16 weeks (Q16W) in patients with nAMD [23]. TENAYA/LUCERNE met their primary endpoint of non-inferior change from baseline in best-corrected visual acuity (BCVA) averaged over weeks 40, 44, and 48 with faricimab up to Q16W versus aflibercept every 8 weeks (Q8W). Anatomical outcomes with faricimab supported the vision gains, with reductions in central subfield thickness (CST), total choroidal neovascularization (CNV) lesion area, and total area of leakage from baseline at the primary endpoint that were comparable with aflibercept [23]. These visual outcomes and anatomical improvements were achieved with extended durability; approximately 80% of patients were on every 12 weeks (Q12W) or Q16W dosing intervals at week 48. Faricimab was well tolerated, with an acceptable safety profile similar to aflibercept. The comparable vision gains and anatomic outcomes with faricimab versus aflibercept were maintained through year 2 of TENAYA and LUCERNE [24]. Based on these findings, faricimab has been approved for the treatment of nAMD in several countries in North America, Europe, and Asia–Pacific.

In this report, we evaluate the 1-year efficacy, durability, and safety of faricimab versus aflibercept in patients from Asian countries (Hong Kong, Japan, Singapore, South Korea, and Taiwan) enrolled in the TENAYA/LUCERNE trials of nAMD, as compared with patients from non-Asian countries.

## Methods

### Study design

The study design and rationale for TENAYA and LUCERNE have been previously described [23]. In brief, TENAYA (ClinicalTrials.gov identifier: NCT03823287) and LUCERNE (NCT03823300) were identically designed, multicenter, randomized, active comparator-controlled, double-masked, parallel-group, 112-week trials conducted at 271 clinical sites worldwide (TENAYA 149 sites in 15 countries, LUCERNE 122 sites in 20 countries). Study protocols were approved by appropriate regulatory authorities, applicable institutional review boards, and ethics committees and were conducted in accordance with the Declaration of Helsinki and principles of Good Clinical Practice. All patients provided written informed consent.

### Participants

Key inclusion criteria were age ≥ 50 years at randomization; treatment-naïve CNV (also referred to as macular neovascularization [25] secondary to nAMD); subfoveal CNV or juxtafoveal or extra foveal CNV, with subfoveal component related to CNV activity, confirmed by fundus fluorescein angiography (FFA), and CNV exudation confirmed on spectral-domain optical coherence tomography (SD-OCT); CNV lesion size ≤ 9 disc areas and CNV component area of ≥ 50% of total lesion area; and Early Treatment Diabetic Retinopathy Study (ETDRS) BCVA 78 − 24 letters (20/32 − 20/320 approximate Snellen equivalent).

### Randomization and masking

Eligible patients were randomized 1:1 to faricimab up to Q16W or aflibercept Q8W using identification numbers assigned by an interactive voice- or web-based response system.

To preserve masking, all patients attended study visits every 4 weeks (Q4W) and received sham procedures at non-active dosing visits. Further details have been described previously [23].

### Treatment protocol

Patients in the aflibercept group received 3 initial intravitreal aflibercept 2.0 mg Q4W doses followed by Q8W dosing to study end in line with international label guidance. After 4 initial intravitreal faricimab 6.0 mg Q4W doses, patients in the faricimab group received Q8W, Q12W, or Q16W dosing based on protocol-defined structural and functional disease activity criteria and treating physician clinical assessment at weeks 20 and 24. Patients with active disease at week 20 received Q8W dosing; patients with active disease at week 24 only received Q12W dosing; patients with no evidence of active disease at weeks 20 and 24 received Q16W dosing. These faricimab regimens were continued up to week 60.

In year 2 of the study, all patients in the faricimab group were scheduled to receive an active dose of faricimab starting at week 60 and were then treated according to a protocol-driven treat-and-extend–based personalized treatment interval (PTI) regimen. In the PTI regimen, dosing intervals could be extended in 4-week increments to a maximum of Q16W, reduced in 4-week or 8-week increments to a minimum of Q8W, or maintained based on disease activity assessments at study drug dosing visits. Study treatment was administered up to week 108, with the final visit at week 112.

As previously described [23], standardized ocular imaging (color fundus photography, FFA, and SD-OCT) was performed at prespecified time points and ocular images were independently assessed by masked evaluators at central reading centers.

### Subgroup analyses and outcome measures

In the present analysis, 1-year outcomes were assessed for the pooled TENAYA/LUCERNE trials for the Asian country and non-Asian country subgroups. The Asian subgroup was defined as patients enrolled in: Hong Kong, Japan, Singapore, South Korea, and Taiwan. The non-Asian country subgroup was defined as patients enrolled in: Argentina, Australia, Austria, Brazil, Bulgaria, Canada, Denmark, France, Germany, Hungary, Israel, Italy, Mexico, the Netherlands, Poland, Portugal, Russia, Spain, Switzerland, Turkey, the United Kingdom, and the United States.

Efficacy and safety endpoints assessed were consistent with prespecified endpoints in the primary analysis [23], and included BCVA change from baseline at the primary endpoint visits (averaged over weeks 40, 44, and 48) and over time; the proportion of patients gaining and avoiding loss of ≥ 15 letters from baseline BCVA up to week 48; the proportion of patients receiving faricimab at Q8W, Q12W, or Q16W intervals at week 48; change from baseline in CST at the primary endpoint visits and over time; and the incidence and severity of ocular and non-ocular adverse events (AEs) up to week 48.

### Statistical analysis

The statistical methods used for efficacy and safety analyses of the Asian country and non-Asian country patient subgroup analyses were consistent with the global TENAYA/LUCERNE patient population and have been described in full previously [23]. Efficacy endpoints measured on a continuous scale were analyzed using a mixed model for repeated measures (MMRM), which was adjusted for treatment group, visit, visit-by-treatment group interaction, baseline variable of interest (continuous), baseline BCVA score (≥ 74, 73–55, or ≤ 54 letters), baseline low-luminance deficit (< 33 or ≥ 33 letters), region (United States, Canada, Asia, and the rest of the world), and study (TENAYA or LUCERNE). An unstructured covariance structure was used. Missing data were implicitly imputed by MMRM. For binary endpoints, weighted proportions were estimated based on Cochran–Mantel–Haenszel (CMH) test stratified by baseline BCVA score (≥ 74, 73–55, or ≤ 54 letters), baseline low-luminance deficit (< 33 or ≥ 33 letters), region (United States and Canada or the rest of the world), and study (TENAYA or LUCERNE).

For the primary analysis, COVID-19–related intercurrent events were handled using a hypothetical strategy where all values were censored after the intercurrent event. For intercurrent events not due to COVID-19, a treatment policy strategy was applied, whereby all observed values were used regardless of occurrence of the intercurrent event. Safety was assessed through descriptive summaries of ocular and systemic AEs, deaths, and ocular assessments up to week 48. AEs were coded per the Medical Dictionary for Regulatory Activities version 23.1 thesaurus and tabulated by System Organ Class and Preferred Term (PT). Ocular assessments were summarized by time point and eye (study vs. fellow eye). No formal statistical comparisons were made between the Asian country and non-Asian country patient subgroups. Efficacy analyses are reported with 95% confidence intervals (CIs). Statistical analyses were performed using SAS version 9.4 (SAS Institute, Inc., Cary, NC).

## Results

### Patient disposition

A total of 1329 treatment-naïve patients were randomized in TENAYA/LUCERNE [23]. Of these, 120 (9.0%) of patients were enrolled in Asia (hereafter referred to as the Asian country subgroup) with 61 patients randomized to faricimab and 59 patients randomized to aflibercept. All patients received ≥ 1 dose of study treatment and were therefore included in both the intention-to-treat and safety analysis populations (Supplementary Information 1A). There were 1209 (91.0%) patients enrolled in the non-Asian countries (hereafter referred to as the non-Asian country subgroup); 604 and 605 of these patients were randomized to faricimab and aflibercept, respectively, and were included in the intention-to-treat population (Supplementary Information 1B). Of these, 1206 (99.8%) patients (603 patients in each of faricimab and aflibercept groups) received ≥ 1 dose of study treatment and were included in the safety analysis population.

The proportion of patients remaining on treatment at week 48 was consistent across the country subgroups and between treatment groups (Asian and non-Asian faricimab groups = 93.4%; Asian aflibercept group = 100%; Non-Asian aflibercept group = 93.9%). The main reasons for study treatment discontinuation among the Asian country subgroup were AEs (1.7% [*n* = 2]), lack of efficacy (0.8% [*n* = 1]), and subject withdrawal (0.8% [*n* = 1]). In the non-Asian country subgroup, the main reasons for study treatment discontinuation were subject withdrawal (2.6% [*n* = 31]), death (1.0% [*n* = 12]), and AEs (0.9% [*n* = 11]).

The mean number of study drug administrations up to week 48 for faricimab and aflibercept was consistent between the Asian and non-Asian country subgroups. The mean ± standard deviation (SD) number of faricimab injections was 6.2 ± 1.01 and 6.4 ± 1.09 for the Asian and non-Asian country subgroups, respectively. The mean ± SD number of aflibercept injections was 7.8 ± 0.57 for the Asian country subgroup and 7.4 ± 1.18 for the non-Asian country subgroup, which was higher than the numbers for faricimab, as expected per the study design. The mean duration of faricimab treatment was 45.6 and 46.3 weeks for the Asian country and non-Asian country subgroups, respectively.

### Baseline demographic and ocular characteristics

Patient demographics and baseline characteristics were generally similar between the Asian country and non-Asian country subgroups and between treatment groups (Table 1). Exceptions to this included age, with the Asian subgroup generally younger than the non-Asian subgroup (mean age 70.9 vs. 76.4 years, respectively), and sex, with fewer females in the Asian country (34/120; 28.3%) versus the non-Asian country subgroup (759/1209; 62.8%). In addition, more patients in the Asian subgroup were current smokers (24.2%) compared with patients in the non-Asian country subgroup (13.9%). Body mass index was lower in the Asian country subgroup with a mean of 24.3 kg/m^2^ compared with 28.2 kg/m^2^ for the non-Asian country subgroup. Most patients in the Asian country subgroup were Japanese (43%), while the majority of the non-Asian country subgroup were white (95%).

**Table 1:** Baseline demographic and ocular characteristics, intent-to-treat population

	Asian country subgroup (*n* = 120)		Non-Asian country subgroup (*n* = 1209)	
	Faricimab 6 mg (*n* = 61)	Aflibercept 2 mg (*n* = 59)	Faricimab 6 mg (*n* = 604)	Aflibercept 2 mg (*n* = 605)
Age (years), mean ± SD^a^	71.1 ± 8.6	70.7 ± 9.0	75.8 ± 8.4	77.0 ± 8.5
Sex, *n* (%)				
Female	15 (24.6%)	19 (32.2%)	379 (62.7%)	380 (62.8%)
Male	46 (75.4%)	40 (67.8%)	225 (37.3%)	225 (37.2%)
Ethnicity or race, *n* (%)^b^				
Hispanic or Latino	0	1 (1.7%)	61 (10.1%)	71 (11.7%)
White	0	0	581 (96.2%)	572 (94.5%)
American Indian or Alaska Native	0	0	2 (0.3%)	2 (0.3%)
Black or African American	0	0	2 (0.3%)	8 (1.3%)
Asian^c^	61 (100%)	59 (100%)	3 (0.5%)	3 (0.5%)
Japanese	26 (42.6%)	26 (44.1%)	0	1 (33.3%)
Korean	16 (26.2%)	19 (32.2%)	1 (33.3%)	0
Taiwanese	11 (18.0%)	8 (13.6%)	0	0
Chinese	8 (13.1%)	6 (10.2%)	0	0
Other Asian	0	0	1 (33.3%)	2 (66.7%)
Asian Indian	0	0	1 (33.3%)	0
Region, *n* (%)				
US and Canada	0	0	317 (52.5%)	316 (52.2%)
Rest of the world^d^	0	0	287 (47.5%)	289 (47.8%)
Asia^e^	61 (100%)	59 (100%)	0	0
Smoking status, *n* (%)				
Never smoked	22 (36.1%)	23 (39.0%)	291 (48.2%)	309 (51.1%)
Previous smoker	25 (41.0%)	21 (35.6%)	226 (37.4%)	215 (35.5%)
Current smoker	14 (23.0%)	15 (25.4%)	87 (14.4%)	81 (13.4%)
BMI, mean ± SD	24.4 ± 3.6	24.1 ± 3.1	28.1 ± 5.7	28.3 ± 5.7
BCVA (ETDRS letters), mean ± SD	59.9 ± 13.6	59.3 ± 13.6	60.0 ± 13.3	60.3 ± 13.1
BCVA categories, *n* (%)				
≥ 74 (20/32 or better)	8 (13.1%)	6 (10.2%)	84 (13.9%)	85 (14.0%)
73–55 (between 20/40 and 20/80)	32 (52.5%)	34 (57.6%)	349 (57.8%)	350 (57.9%)
≤ 54 (20/80 or worse)	21 (34.4%)	19 (32.2%)	171 (28.3%)	170 (28.1%)
CST (ILM-RPE) (µm), mean ± SD	336.5 ± 133.1	334.3 ± 104.2	358.8 ± 120.8	359.8 ± 120.6
Intraocular pressure (mm Hg), mean ± SD	14.1 ± 3.2	14.1 ± 2.9	15.1 ± 2.9	14.9 ± 3.0
Time since AMD diagnosis, *n* (%)				
≤ 1 month	35 (57%)	34 (58%)	434 (72%)	420 (69%)
> 1 month	22 (36%)	22 (37%)	140 (23%)	162 (27%)
Phakic, *n* (%)	45 (73.8%)	48 (81.4%)	338 (56.0%)	321 (53.1%)
Presence of IRF, *n* (%)	19 (31.1%)	17 (28.8%)	269 (44.5%)	294 (48.6%)
Presence of SRF, *n* (%)	45 (73.8%)	47 (79.7%)	392 (64.9%)	400 (66.1%)
CNV location by FFA, *n* (%)				
Subfoveal	35 (57.4%)	28 (47.5%)	375 (62.1%)	349 (57.7%)
Juxtafoveal	14 (23.0%)	19 (32.2%)	147 (24.3%)	153 (25.3%)
Extrafoveal	11 (18.0%)	11 (18.6%)	72 (11.9%)	88 (14.5%)
CNV lesion type by FFA, *n* (%)				
Classic/predominantly classic	12 (19.7%)	15 (25.4%)	193 (32.0%)	202 (33.4%)
Occult/minimally classic/PCV	43 (70.5%)	37 (62.7%)	378 (62.6%)	352 (58.2%)
RAP	5 (8.2%)	6 (10.2%)	23 (3.8%)	36 (6.0%)
Missing	1 (1.6%)	1 (1.7%)	10 (1.7%)	15 (2.5%)
Total area of CNV lesion by FFA (mm^2^), mean ± SD	3.8 ± 4.0	4.4 ± 4.6	4.8 ± 4.8	4.4 ± 4.2
PCV status per ICGA, *n* (%)^f^	8 (18.6%)	8 (22.9%)	0	0

^a^Age at randomization

^b^Not all race categories are listed; therefore, the sum of proportions shown may not equal 100%

^c^Percentages for Asian race subgroups are based on the number of Asian patients in each treatment group

^d^Non-Asian includes all subjects from Argentina, Australia, Austria, Brazil, Bulgaria, Canada, Denmark, France, Germany, Hungary, Israel, Italy, Mexico, the Netherlands, Poland, Portugal, Russia, Spain, Switzerland, Turkey, United Kingdom, and United States

^e^Asia includes Hong Kong, Japan, Singapore, South Korea, and Taiwan

^f^For the Asian country subgroup: faricimab *n* = 43 and aflibercept *n* = 35; for the non-Asian country subgroup: faricimab *n* = 124; aflibercept *n* = 109

*AMD* age-related macular degeneration, *BCVA* best-corrected visual acuity, *BMI* body mass index, *CNV* choroidal neovascularization, *CST* central subfield thickness, *ETDRS* Early Treatment Diabetic Retinopathy Study, *FFA* fundus fluorescein angiography, *ICGA* indocyanine green angiography, *ILM* internal limiting membrane, *IRF* intraretinal fluid, *PCV* polypoidal choroidal vasculopathy, *RAP* retinal angiomatous proliferation, *RPE* retinal pigment epithelium, *SD* standard deviation, *SRF* subretinal fluid

Baseline ocular characteristics were generally well balanced between treatment groups within each subgroup (Table 1). Mean baseline BCVA was 59.6 ETDRS letters for the Asian country subgroup and 60.2 letters for non-Asian country subgroup. Most patients (55.0% and 57.8% in the Asian country and non-Asian country subgroups, respectively) had a baseline BCVA of 73–55 letters (Snellen equivalent 20/40–20/80). Mean baseline CST was lower in the Asian country subgroup compared with the non-Asian country subgroup (335.4 µm and 359.3 µm, respectively). The proportion of patients with presence of intraretinal fluid was lower in the Asian country versus the non-Asian country subgroup (30.0% and 46.6%, respectively). The frequency of patients with classic/predominantly classic CNV lesion type in the Asian country subgroup (22.5%) was lower than in the non-Asian country subgroup (32.7%). In the Asian country subgroup, 66.7% of patients had occult/minimally classic/PCV lesions, which was higher than in the non-Asian country subgroup (60.4%). PCV was assessed on optional indocyanine green angiography (ICGA) in 65.0% (78/120) of the Asian country subgroup and 19.3% (233/1209) of the non-Asian country subgroup; the Asian country subgroup had a higher proportion of patients with PCV (20.5% [16/78]), compared with the non-Asian country subgroup (0.0% [0/233]).

### Vision outcomes

**Fig. 1:**
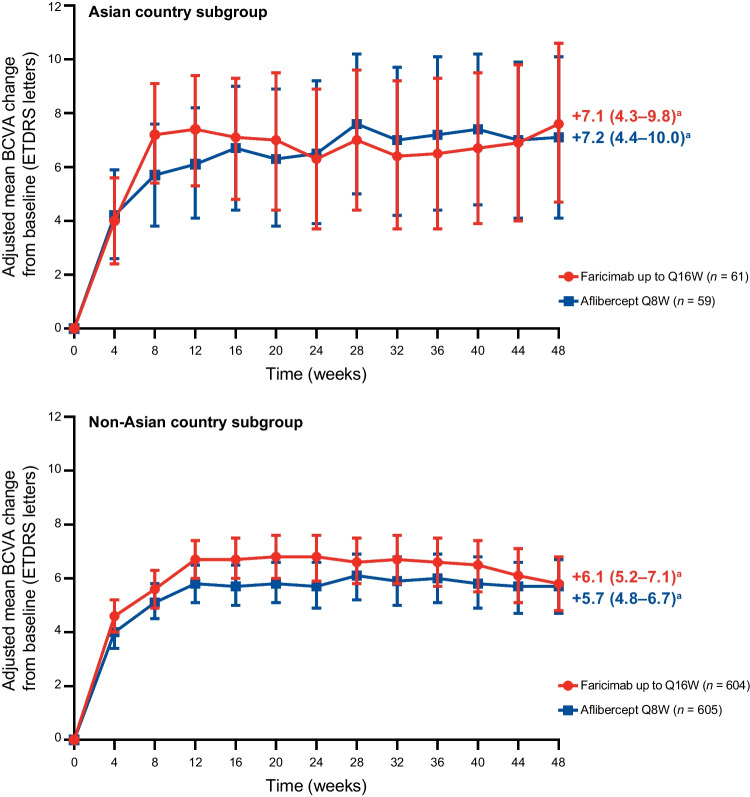
Adjusted mean change in BCVA (ETDRS letters) from baseline up to week 48 in the pooled TENAYA/LUCERNE Asian country and non-Asian country subgroups. Results are based on MMRM analysis; missing data were implicitly imputed by MMRM. Error bars represent 95% CIs. ^a^Adjusted mean BCVA change from baseline was averaged over weeks 40, 44, and 48. *BCVA* best-corrected visual acuity, *ETDRS* Early Treatment Diabetic Retinopathy Study, *MMRM* mixed model for repeated measures, *Q8W* every 8 weeks, *Q16W* every 16 weeks

In the global population, both TENAYA and LUCERNE met their primary endpoint of non-inferiority in change from baseline in BCVA in the study eye at the primary endpoint visits (averaged over weeks 40, 44, and 48) with faricimab up to Q16W compared with aflibercept Q8W [23]. In the Asian country subgroup, adjusted mean gains in BCVA at the primary endpoint visits were 7.1 ETDRS letters (95% CI, 4.3 to 9.8) in the faricimab group and 7.2 letters (4.4–10.0) in the aflibercept group (Fig. 1). In the non-Asian country subgroup, vision gains were 6.1 ETDRS letters (5.2–7.1) in the faricimab group and 5.7 letters (4.8–6.7) in the aflibercept group. Similar increases in BCVA from baseline were observed up to week 48 between treatment groups in both the Asian country and non-Asian country subgroups (Fig. 1).

The proportion of patients among the Asian country subgroup that gained ≥ 15 letters from baseline at week 48 was numerically higher with faricimab (31.7% [95% CI, 20.1–43.2%]) versus aflibercept (19.8% [10.1–29.5%) (Supplementary Information 2). Corresponding proportions among the non-Asian country subgroup were similar between faricimab and aflibercept (20.7% [17.4–24.0%] vs. (21.6% [18.3–25.0%], respectively). Additionally, the proportion of patients in the faricimab groups that gained ≥ 15 letters from baseline at week 48 was numerically higher in the Asian compared with the non-Asian subgroup. More than 90% of patients in the Asian country (96.1% and 96.5% in faricimab and aflibercept, respectively) and non-Asian country (94.7% and 95.0% in faricimab and aflibercept, respectively) subgroups avoided a loss of ≥ 15 letters in BCVA from baseline at week 48 (Supplementary Information 3).

### Durability and anatomic outcomes

**Fig. 2:**
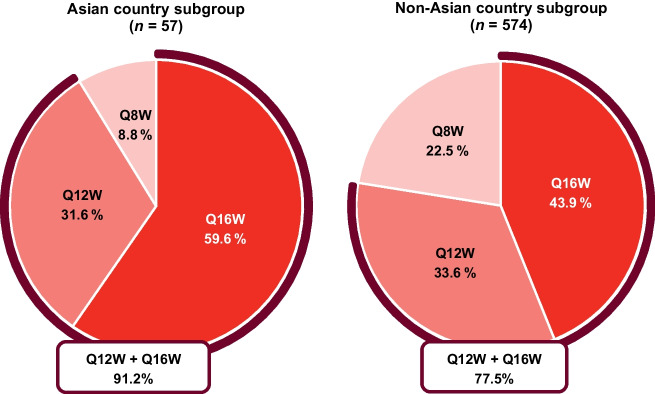
Proportion of patients in the faricimab group who completed week 48 treatment on Q8W, Q12W, or Q16W fixed dosing in the pooled TENAYA/LUCERNE Asian country and non-Asian country subgroups. Analyses included patients in the faricimab groups who had not discontinued the study at the week 48 visit. The treatment interval at week 48 was defined as the treatment interval decision made at that visit. *Q8W* every 8 weeks, *Q12W* every 12 weeks, *Q16W* every 16 weeks

Similar to the global trial population, faricimab showed strong durability in the Asian country subgroup, with > 90% of patients achieving Q12W or Q16W dosing at week 48 (Fig. 2). The proportion of faricimab-treated patients achieving extended dosing (receiving ≥ Q12W) in the Asian country subgroup was numerically higher (~ 91%) but consistent with the non-Asian country subgroup (~ 78%).

**Fig. 3:**
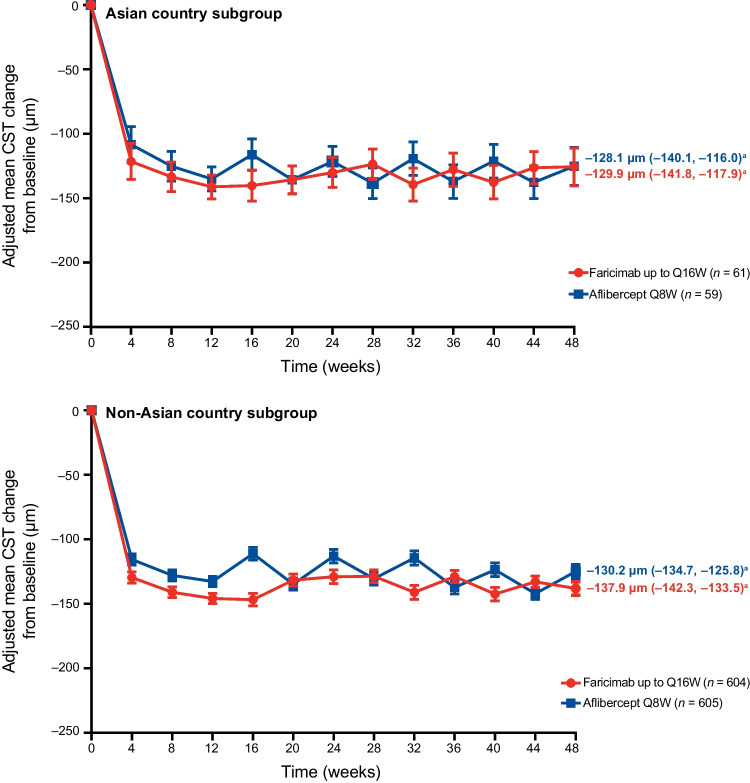
Adjusted mean change in CST from baseline up to week 48 in the pooled TENAYA/LUCERNE Asian country and non-Asian country subgroups. Results are based on MMRM analysis; missing data were implicitly imputed by MMRM. ^a^Adjusted mean CST change from baseline was averaged over weeks 40, 44, and 48. *CST* central subfield thickness, *MMRM* mixed model for repeated measures, *Q8W* every 8 weeks, *Q16W* every 16 weeks

CST reductions from baseline at all time points up to week 48 were similar between the Asian country and non-Asian country subgroups. Mean reductions in CST were numerically greater with faricimab compared with aflibercept during the initial matched-dosing period (up to week 12). The adjusted mean CST change from baseline at the primary endpoint visits for patients from Asian countries was –129.9 µm (95% CI, –141.8 to − 117.9) for faricimab and –128.1 µm (–140.1 to − 116.0) for aflibercept. Corresponding reductions for patients from non-Asian countries were –137.9 µm (–142.3 to − 133.5) for faricimab, which was numerically greater than reductions for aflibercept (–130.2 µm [–134.7 to − 125.8]) (Fig. 3).

### Safety outcomes

Faricimab was well tolerated up to week 48, with no clinically relevant difference in AE incidence observed between the Asian country and non-Asian country subgroups or between treatment groups (Table 2). The incidence of AEs leading to study treatment discontinuation up to week 48 was low and generally similar between the Asian country (faricimab *n* = 2 [3.3%], aflibercept *n* = 0 [0%]) and the non-Asian country subgroups (faricimab *n* = 9 [1.5%], aflibercept *n* = 4 [0.7%]). The incidence of ocular AEs in the study eye across the faricimab and aflibercept groups was numerically lower in the Asian country (faricimab *n* = 18 [29.5%], aflibercept *n* = 15 [25.4%]) versus the non-Asian country subgroup (faricimab *n* = 236 [39.1%], aflibercept *n* = 231 [38.3%]). The most common ocular AEs in the study eye by PT in faricimab-treated patients from Asian countries (≥ 2% in the faricimab group) were dry eye (4.9% [*n* = 3]), intercepted medication error (3.3% [*n* = 2]), intraocular pressure increased (3.3% [*n* = 2]), vitreous floaters (3.3% [*n* = 2]), retinal pigment epithelium (RPE) tear (3.3% [*n* = 2]), and posterior capsule opacification (3.3% [*n* = 2]). In the non-Asian country subgroup, the most common ocular AEs in the study eye by PT in the faricimab group were conjunctival hemorrhage (7.3% [*n* = 44]), worsening of nAMD (6.3% [*n* = 38]), vitreous detachment (3.6% [*n* = 22]), cataract (3.2% [*n* = 19]), vitreous floaters (3.0% [*n* = 18]), RPE tear (2.8% [*n* = 17]), eye pain (2.7% *n* = 16]), and intraocular pressure increased (2.5% [*n* = 15]). The incidence of ocular serious AEs reported was low and similar between faricimab and aflibercept for both the Asian country subgroup (faricimab *n* = 1 [1.6%], aflibercept *n* = 1 [1.7%]) and the non-Asian country subgroup (faricimab *n* = 10 [1.7%], aflibercept *n* = 12 [2.0%]).

**Table 2:** Summary of key AEs up to week 48, safety evaluable population

	Asian country subgroup (*n* = 120)		Non-Asian country subgroup (*n* = 1206)	
	Faricimab 6 mg (*n* = 61)	Aflibercept 2 mg (*n* = 59)	Faricimab 6 mg (*n* = 603)	Aflibercept 2 mg (*n* = 603)
Total number of AEs^a^	120	88	1550	1570
Total number of SAEs^a^	8	5	107	184
Patients with ≥ 1 ocular AE^b^	18 (29.5%)	15 (25.4%)	236 (39.1%)	231 (38.3%)
Patients with ≥ 1 ocular SAE^b^	1 (1.6%)	1 (1.7%)	10 (1.7%)	12 (2.0%)
Patients with ≥ 1 non-ocular AE	31 (50.8%)	28 (47.5%)	315 (52.2%)	335 (55.6%)
Patients with ≥ 1 non-ocular SAE	6 (9.8%)	3 (5.1%)	62 (10.3%)	79 (13.1%)
Patients with ≥ 1 treatment-related ocular AE^b^	4 (6.6%)	2 (3.4%)	15 (2.5%)	15 (2.5%)
Patients with ≥ 1 treatment-related ocular SAE^b^	1 (1.6%)	0	7 (1.2%)	1 (0.2%)
Patients with ≥ 1 ocular AE of special interest^b,c^	1 (1.6%)	1 (1.7%)	7 (1.2%)	11 (1.8%)
Patients with ≥ 1 AE of IOI (excluding endophthalmitis) ^b^^,d^	1 (1.6%)	0	12 (2.0%)	8 (1.3%)
Iridocyclitis	1 (1.6%)	0	2 (0.3%)	2 (0.3%)
Iritis	0	0	3 (0.5%)	2 (0.3%)
Uveitis^e^	0	0	2 (0.3%)	2 (0.3%)
Vitritis	0	0	3 (0.5%)	1 (0.2%)
Chorioretinitis	0	0	1 (0.2%)	0
Keratic precipitates	0	0	1 (0.2%)	0
Post-procedural inflammation	0	0	0	1 (0.2%)
Patients with ocular SAE known to be associated with anti-VEGF^b^				
Endophthalmitis	0	0	0	1 (0.2%)
Rhegmatogenous retinal detachment	0	0	0	0
Retinal tear	0	0	0	0
Retinal pigment epithelial tear	1 (1.6%)	0	3 (0.5%)	0
Intraocular pressure increased	0	0	1 (0.2%)	0
Traumatic cataract	0	0	0	0
Retinal vasculitis and retinal occlusive events^b^				
Retinal vasculitis	0	0	0	0
Retinal vein occlusion	0	0	0	0
Retinal artery occlusion	0	0	0	0
Retinal artery embolism	0	0	1 (0.2%)^f^	0
Patients with ≥ 1 APTC^g^ event	0	0	7 (1.2%)	6 (1.0%)
Death	0	0	2 (0.3%)	3 (0.5%)
Non-fatal myocardial infarction	0	0	3 (0.5%)	2 (0.3%)
Non-fatal stroke	0	0	2 (0.3%)	1 (0.2%)

Data are *n* or *n* (%)

^a^Total number of AEs and SAEs includes non-ocular and ocular events in the study or fellow eye

^b^Ocular AEs and SAEs in the study eye only

^c^AEs of special interest includes events associated with severe IOI, events that result in a BCVA decrease of 30 letters or more for more than 1 h, or events that require intervention or surgery to prevent permanent vision loss. For frequency counts by Preferred Term, multiple occurrences of the same AE in an individual are counted only once. Includes AEs with onset up to day 349 (last day of week 48 analysis visit window)

^d^Includes serious and non-serious IOI events

^e^Severe IOI events are reported; all other events were mild or moderate

^f^Hollenhorst plaque

^g^APTC events were adjudicated by an external committee; all other events were investigator reported

*AE* adverse event, *APTC* Antiplatelet Trialists’ Collaboration, *BCVA* best-corrected visual acuity, *IOI* intraocular inflammation, *SAE* serious adverse event, *VEGF* vascular endothelial growth factor

The incidence of intraocular inflammation (IOI) reported for both studies was low. In faricimab-treated patients, the incidence of IOI in the study eye in the Asian country subgroup was 1.6% (*n* = 1), which was similar to that in the non-Asian country subgroup (2.0% [*n* = 12]). There were no cases of IOI in the study eye reported for aflibercept-treated patients in the Asian subgroup, while the incidence of IOI for aflibercept-treated patients in the non-Asian country subgroup was 1.3% (*n* = 8). There were no cases of retinal vasculitis or IOI associated with retinal occlusive events reported in either study. There was a single asymptomatic retinal artery embolism, a Hollenhorst plaque, reported for a patient receiving faricimab in the non-Asian country subgroup; the event was not considered to be treatment related by the investigator.

The incidence of non-ocular AEs was similar between the treatment groups in both the Asian country and non-Asian country subgroups. Specifically, the incidence was 50.8% (*n* = 31) and 47.5% (*n* = 28) for faricimab and aflibercept, respectively, in the Asian country subgroup, and 52.2% (*n* = 315) and 55.6% (*n* = 335) for faricimab and aflibercept, respectively, in the non-Asian country subgroup. In the Asian country subgroup, there were no externally adjudicated Antiplatelet Trialists’ Collaboration (APTC)-defined AEs observed. In the non-Asian country subgroup, the incidence of externally adjudicated APTC-defined AEs was low (1.2% [*n* = 7] for faricimab and 1.0% [*n* = 6] for aflibercept).

## Discussion

This subgroup analysis of the TENAYA/LUCERNE trials is the first to evaluate the efficacy, durability, and safety of faricimab in patients from Asian and non-Asian countries. The results indicate that visual and anatomical outcomes with faricimab up to Q16W were similar to those seen with aflibercept Q8W in both the Asian country and non-Asian country subgroups. Efficacy outcomes were achieved with the majority of patients on extended dosing of faricimab (≥ Q12W; > 90% and ~ 78% in the Asian country and non-Asian country subgroups, respectively) at week 48.

In keeping with the global primary analyses [23], similar BCVA gains from baseline at the primary endpoint visits were observed between the Asian country and non-Asian country subgroups for the faricimab and aflibercept groups. Initial rapid BCVA gains persisted up to week 48 in both the Asian country and non-Asian country subgroups between treatment groups, and nearly all patients avoided losing ≥ 15 BCVA letters from baseline up to week 48. A numerically higher proportion of patients from Asian compared with non-Asian countries gained ≥ 15 letters from baseline at week 48 with faricimab.

CST reductions supported the visual outcomes and were similar between the Asian country and non-Asian country subgroups. Between treatment groups, meaningful and comparable reductions in CST from baseline were observed at the primary endpoint visits and over time up to week 48. Visual and anatomical outcomes with faricimab were achieved with extended durability, with a large majority of patients from Asian and non-Asian countries receiving faricimab dosing of ≥ Q12W. Of note, more patients from Asian countries were receiving ≥ Q12W dosing (91.2% vs. 77.5%) and fewer were on Q8W dosing (8.8% vs. 22.5%) compared with patients from non-Asian countries. This result, in addition to more patients from Asian versus non-Asian countries gaining ≥ 15 letters from baseline at week 48 with faricimab, suggests the potential for improved vision outcomes with faricimab in the Asian country subgroup. Based on previous findings of improved visual outcomes in patients with PCV following anti-VEGF therapy, a possible explanation for these results is the higher proportion of patients with PCV in the Asian country subgroup (20.5% [16/78]) versus the non-Asian country subgroup (0.0% [0/233]) [15, 26, 27]. However, these findings should be interpreted with caution given the small sample size in the Asian country subgroup.

Importantly, faricimab was well tolerated in the Asian country subgroup, with an acceptable safety profile that was similar to that of aflibercept and consistent with the non-Asian country subgroup, which is in accordance with the safety findings reported in the global TENAYA/LUCERNE results [23]. The risk of IOI and APTC events was low and similar between patients from Asian and non-Asian countries. There were no cases of retinal vasculitis or IOI associated with retinal occlusive events reported in either study.

An inherent limitation of this analysis is the low number of patients in the Asian country subgroup (*n* = 120) compared with the non-Asian country subgroup (*n* = 1209). Consequently, results observed for the Asian country subgroup should be interpreted with appropriate caution, and future studies enrolling larger numbers of patients from Asian countries are needed. It is noteworthy that the potential effect of PCV subtype on outcomes with faricimab could not be analyzed, given that PCV (as assessed by ICGA) was reported in approximately 20% of patients in the Asian country subgroup, whereas there was no PCV reported in the non-Asian country subgroup. Additionally, in TENAYA/LUCERNE, ICGA was an optional procedure unlike FFA, which was protocol-mandated. Previous studies suggest that anti-VEGF therapy with aflibercept and ranibizumab is safe and efficacious for the treatment of PCV [28, 29]. The potential impact of PCV on outcomes with faricimab will be analyzed in the SALWEEN study (ISRCTN69073386), which will evaluate the efficacy, durability, and safety of faricimab in patients with PCV. TENAYA/LUCERNE were not designed to compare the durability of faricimab relative to aflibercept, which was administered according to a fixed Q8W regimen, as per international label guidance. nAMD is a chronic and progressive disease with long-term effects on vision and as such, the 1-year follow-up period in the current report is short. The long-term follow-up study AVONELLE-X (NCT04777201) will further inform faricimab’s durability and long-term effects.

In summary, results from this subgroup analysis of year 1 results from TENAYA/LUCERNE suggest that faricimab up to Q16W offers vision and anatomical benefits and safety outcomes that are generally similar to aflibercept Q8W and consistent between the Asian and non-Asian countries. In addition, the extended durability up to Q16W with faricimab observed in the global TENAYA/LUCERNE trials was also observed in this subgroup analysis. Disease control offered by dual Ang-2 and VEGF pathway inhibition with faricimab may improve outcomes in patients from Asian countries by allowing extending dosing over that currently achievable with VEGF monotherapies.

### Supplementary information

Below is the link to the electronic supplementary material.

Supplementary file1 (PDF 770 KB)

Supplementary file2 (PDF 833 KB)

Supplementary file3 (PDF 828 KB)

## Data Availability

For eligible studies, qualified researchers may request access to individual patient-level clinical data through a data request platform. At the time of writing, this request platform is Vivli (https://vivli.org/ourmember/roche/). For up-to-date details on Roche's Global Policy on the Sharing of Clinical Information and how to request access to related clinical study documents, see here (https://go.roche.com/data_sharing). Anonymized records for individual patients across more than one data source external to Roche cannot, and should not, be linked due to a potential increase in risk of patient re-identification.
