# Supplementary material for: Efficacy, durability, and safety of faricimab in patients from Asian countries with neovascular age-related macular degeneration: 1-Year subgroup analysis of the TENAYA and LUCERNE trials
Source: Graefes Arch Clin Exp Ophthalmol. 2023 Jun 9;261(11):3125–37. doi: 10.1007/s00417-023-06071-8 (PMC10251323; doi:10.1007/s00417-023-06071-8)
Supplement: Supplementary file 1 — Supplementary file1 (PDF 770 KB) [file 417_2023_6071_MOESM1_ESM.pdf]

Online Resource 1 Patient flow diagrams for the Asian country (a) and non-Asian country (b) subgroups of the pooled TENAYA/LUCERNE trials. *Q8W* every 8 weeks, *Q16W* every 16 weeks.

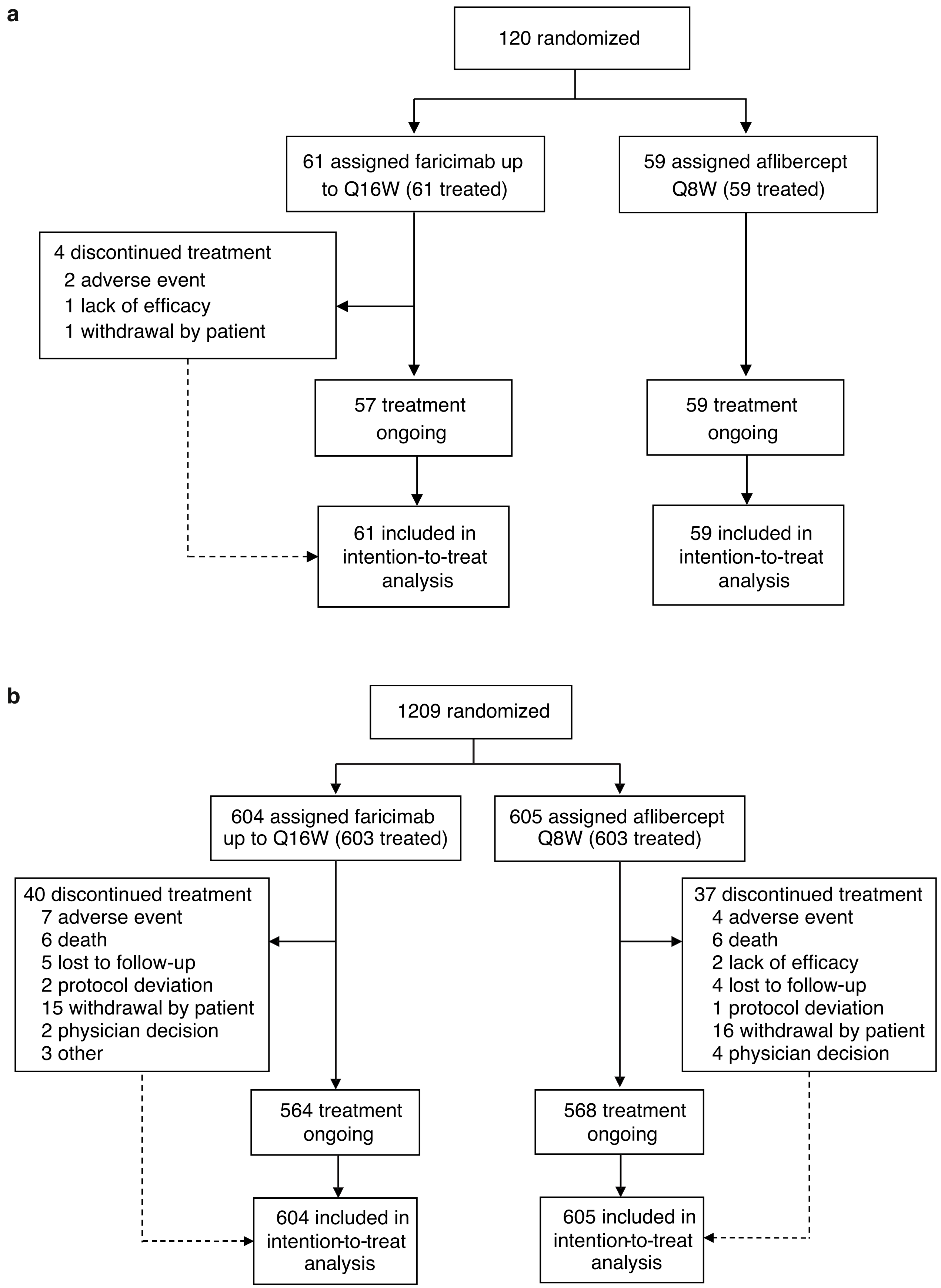
a
120 randomized
61 assigned faricimab up to Q16W (61 treated)
59 assigned aflibercept Q8W (59 treated)
4 discontinued treatment
2 adverse event
1 lack of efficacy
1 withdrawal by patient
57 treatment ongoing
59 treatment ongoing
61 included in intention-to-treat analysis
59 included in intention-to-treat analysis
b
1209 randomized
604 assigned faricimab up to Q16W (603 treated)
605 assigned aflibercept Q8W (603 treated)
40 discontinued treatment
7 adverse event
6 death
5 lost to follow-up
2 protocol deviation
15 withdrawal by patient
2 physician decision
3 other
37 discontinued treatment
4 adverse event
6 death
2 lack of efficacy
4 lost to follow-up
1 protocol deviation
16 withdrawal by patient
4 physician decision
564 treatment ongoing
568 treatment ongoing
604 included in intention-to-treat analysis
605 included in intention-to-treat analysis
