# Supplementary material for: Efficacy, durability, and safety of faricimab in patients from Asian countries with neovascular age-related macular degeneration: 1-Year subgroup analysis of the TENAYA and LUCERNE trials
Source: Graefes Arch Clin Exp Ophthalmol. 2023 Jun 9;261(11):3125–37. doi: 10.1007/s00417-023-06071-8 (PMC10251323; doi:10.1007/s00417-023-06071-8)
Supplement: Supplementary file 2 — Supplementary file2 (PDF 833 KB) [file 417_2023_6071_MOESM2_ESM.pdf]

Online Resource 2 Proportion of patients gaining ≥15 BCVA letters up to week 48 in the pooled TENAYA/LUCERNE Asian country and non-Asian country subgroups. Weighted proportion of patients in each group was estimated using the CMH method. Error bars represent 95% CIs. *BCVA* best-corrected visual acuity, *CI* confidence interval, *CMH* Cochran–Mantel–Haenszel, *Q8W* every 8 weeks, *Q16W* every 16 weeks.

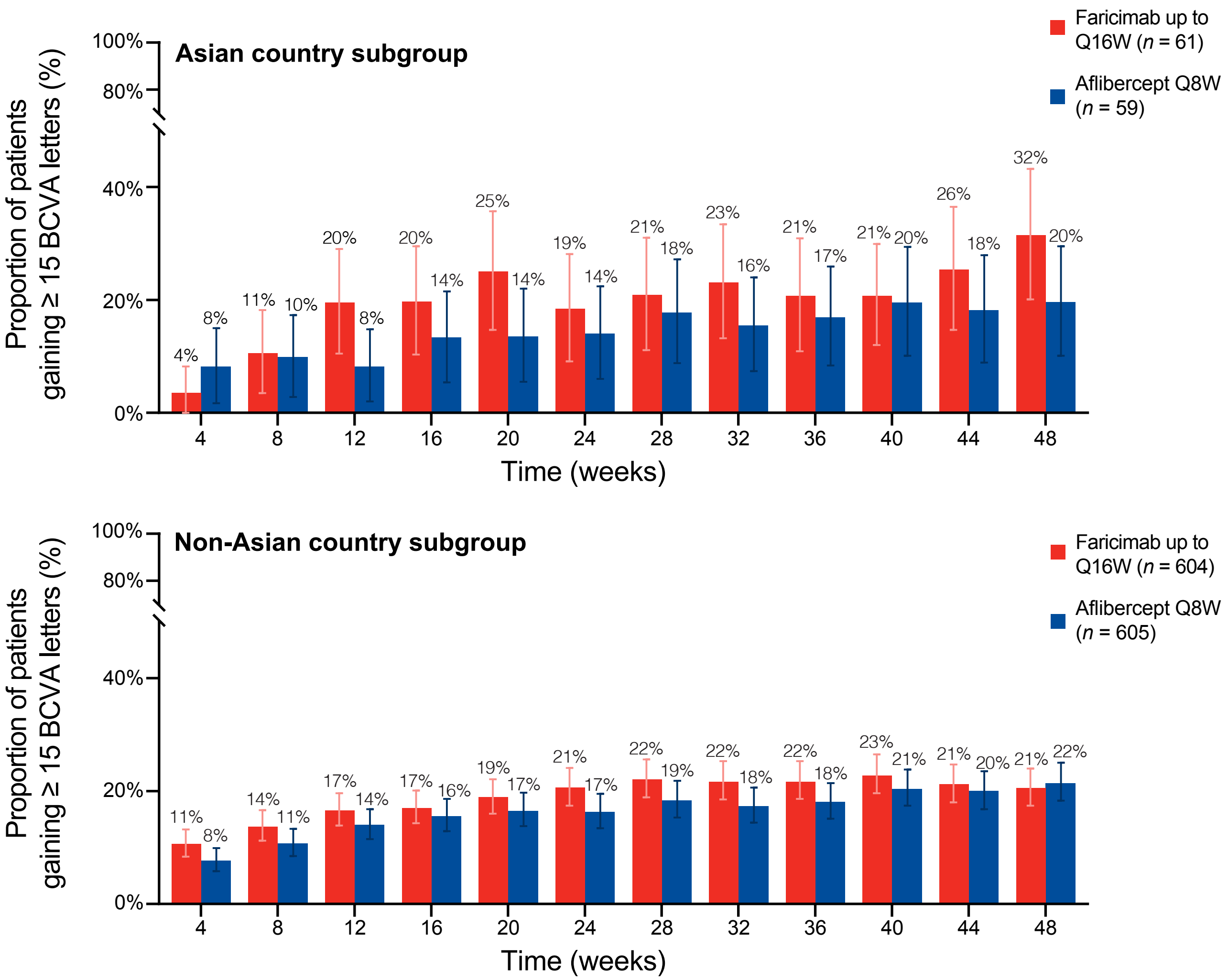

Asian country subgroup
Faricimab up to Q16W (n = 61)
Aflibercept Q8W (n = 59)
Proportion of patients gaining ≥ 15 BCVA letters (%)
100%
80%
40%
20%
0%
4% 8%
11% 10%
20% 8%
20% 14%
25% 14%
19% 14%
21% 18%
23% 16%
21% 17%
21% 20%
26% 18%
32% 20%
4 8 12 16 20 24 28 32 36 40 44 48
Time (weeks)
Non-Asian country subgroup
Faricimab up to Q16W (n = 604)
Aflibercept Q8W (n = 605)
Proportion of patients gaining ≥ 15 BCVA letters (%)
100%
80%
40%
20%
0%
11% 8%
14% 11%
17% 14%
17% 16%
19% 17%
21% 17%
22% 19%
22% 18%
22% 18%
23% 21%
21% 20%
21% 22%
4 8 12 16 20 24 28 32 36 40 44 48
Time (weeks)
