# Supplementary material for: Efficacy, durability, and safety of faricimab in patients from Asian countries with neovascular age-related macular degeneration: 1-Year subgroup analysis of the TENAYA and LUCERNE trials
Source: Graefes Arch Clin Exp Ophthalmol. 2023 Jun 9;261(11):3125–37. doi: 10.1007/s00417-023-06071-8 (PMC10251323; doi:10.1007/s00417-023-06071-8)
Supplement: Supplementary file 3 — Supplementary file3 (PDF 828 KB) [file 417_2023_6071_MOESM3_ESM.pdf]

Online Resource 3 Proportion of patients avoiding loss of ≥15 BCVA letters up to week 48 in the pooled TENAYA/LUCERNE Asian country and non-Asian country subgroups. Weighted proportion of patients in each group was estimated using the CMH method. Error bars represent 95% CIs. *BCVA* best-corrected visual acuity, *CI* confidence interval, *CMH* Cochran–Mantel–Haenszel, *Q8W* every 8 weeks, *Q16W* every 16 weeks.

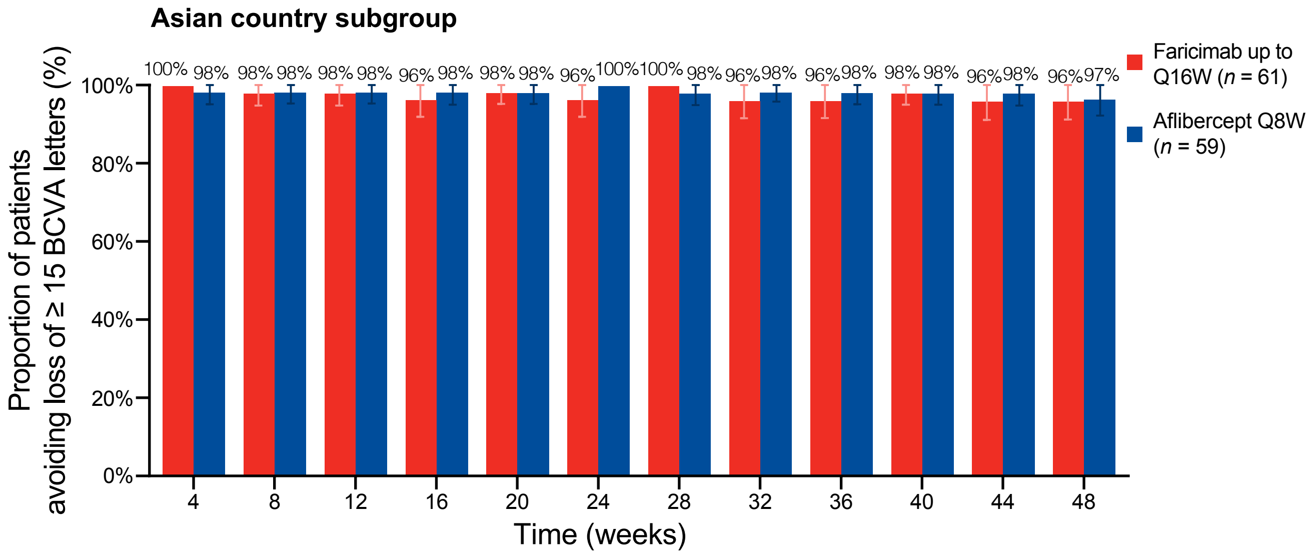

Asian country subgroup
Proportion of patients avoiding loss of ≥ 15 BCVA letters (%)
100%
80%
60%
40%
20%
0%
100% 98% 98% 98% 98% 98% 96% 98% 98% 98% 96% 100% 100% 98% 96% 98% 96% 98% 98% 98% 96% 98% 96% 97%
4 8 12 16 20 24 28 32 36 40 44 48
Time (weeks)
Faricimab up to Q16W (n = 61)
Aflibercept Q8W (n = 59)

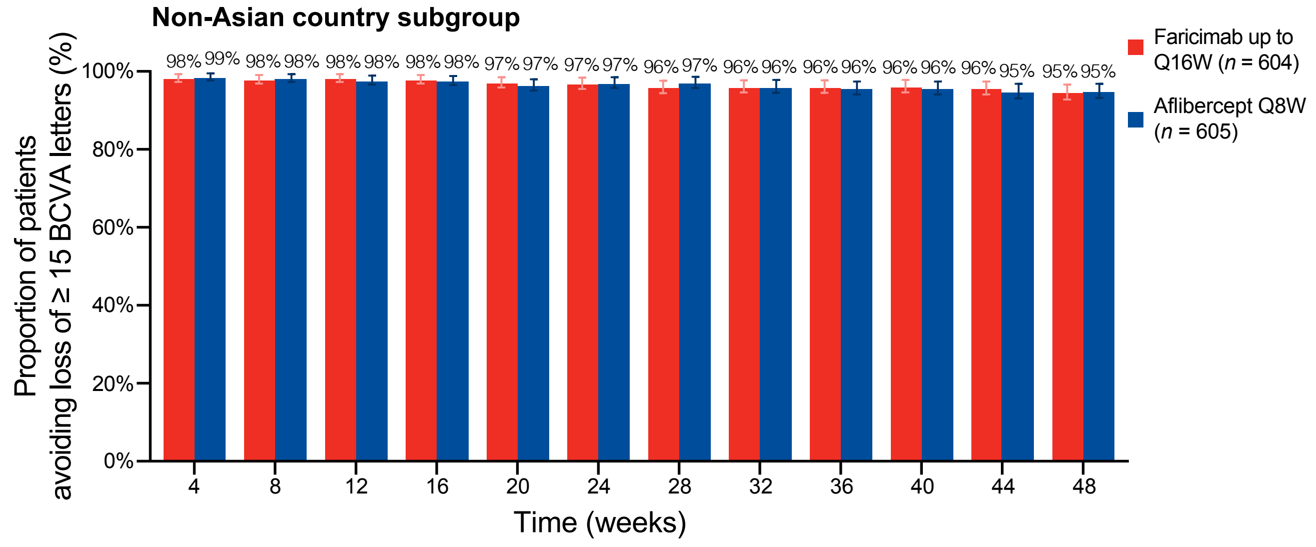

Non-Asian country subgroup
Proportion of patients avoiding loss of ≥ 15 BCVA letters (%)
100%
80%
60%
40%
20%
0%
98% 99% 98% 98% 98% 98% 98% 98% 97% 97% 97% 97% 96% 97% 96% 96% 96% 96% 96% 96% 96% 95% 95% 95%
4 8 12 16 20 24 28 32 36 40 44 48
Time (weeks)
Faricimab up to Q16W (n = 604)
Aflibercept Q8W (n = 605)
